# Long-term impact of the 10-valent pneumococcal conjugate vaccine on nonvaccine pneumococcal serotypes: implications for practice and surveillance

**DOI:** 10.36416/1806-3756/e20250400

**Published:** 2025-11-19

**Authors:** Marcos Brum, Luiza Fernandes Xavier, Gabriela Bastian, Paula Barros de Barros, Eduardo Herter, Marina Pietá, Camila Machado, Frederico Friedrich, Marcelo C Scotta, Leonardo Araujo Pinto

**Affiliations:** 1. Escola de Medicina, Pontifícia Universidade Católica do Rio Grande do Sul - PUCRS - Porto Alegre (RS) Brasil.

## BACKGROUND

Pneumonia remains one of the leading causes of death in children under five years of age worldwide. It is estimated that approximately 700,000 deaths occur annually, and *Streptococcus pneumoniae* is the bacterial agent that is most frequently implicated. The risk is even greater among children with preexisting cardiovascular or respiratory diseases, which makes prevention essential. In this context, pneumococcal conjugate vaccines (PCVs) have radically changed the landscape of public health. The first PCV, the 7-valent PCV, was introduced in 2000, followed by broader formulations such as the 10-valent PCV (PCV10), the 13-valent PCV (PCV13), the 15-valent PCV, and the 20-valent PCV. In Brazil, PCV10 was incorporated into the Brazilian National Immunization Program in 2010, covering ten serotypes but not including 19A.^1^ ^,^ ^2^

**Figure 1:**
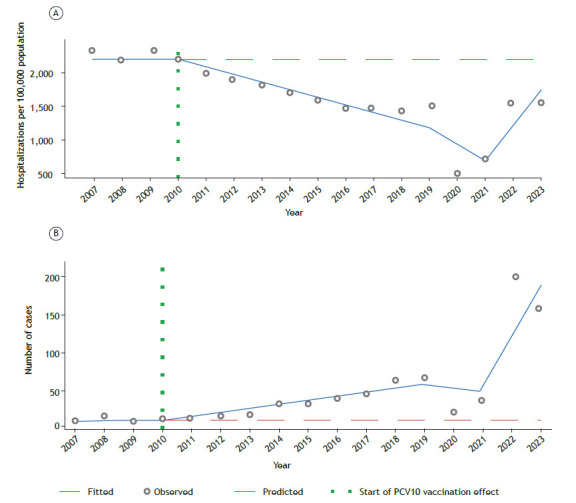
Long-term impact of the 10-valent pneumococcal conjugate vaccine (PCV10) on nonvaccine pneumococcal serotypes. In A, hospitalizations for pneumonia. In B, absolute cases of *Streptococcus pneumonia* serotype 19A.

In the years following the introduction of PCV10, the results were remarkable: according to the Information Technology Department of the Brazilian Unified Health Care System, hospitalizations for pneumonia in children dropped from 2,157.5 to 1,441.5 per 100,000 population between 2010 and 2018 (a reduction of 33.2%). This clearly demonstrates the short-term positive impact of the vaccine. However, the trajectory did not remain linear. Starting in 2019, a reversal trend emerged, culminating with rates of 1,553.7 per 100,000 population in 2023, i.e., a 7.8% increase in comparison with the lowest rate observed in 2018 (Figure 1A). 

## WHAT EXPLAINS THIS CHANGE?

The phenomenon of serotype replacement is the main candidate. When the vaccine dramatically reduces the targeted serotypes, other serotypes find space to spread.^3^

Serotype 19A became the most relevant in Brazil after the introduction of PCV10. Epidemiological analysis showed an average annual increase of 6.06 cases of 19A, with statistical significance (95% CI, 1.39-10.73; p < 0.01; Figure 1B). In practical terms, this means that, even when vaccinated, many children remained vulnerable to severe pneumonia, now caused by a serotype not included in the formulation adopted in the country. 

This pattern is not unique to Brazil. In other Latin American countries, an increase in serotype 19A has also been documented following the adoption of PCV10.^4^ ^,^ ^5^ In places such as the United States, England, and Wales, a similar trajectory of 19A growth was observed, often associated with complicated pneumonia and penicillin resistance. The difference is that in those countries, the introduction of PCV13, which includes serotype 19A, led to significant declines in its prevalence. In the United States, there was a reduction of approximately 40% among children under five years of age. In Israel, the incidence of invasive respiratory disease caused by 19A fell from 5 to 1.6 per 100,000 population after the introduction of PCV13. These data reinforce that the choice of vaccine formulation has a direct impact on epidemiological dynamics and clinical outcomes.^2^ ^,^ ^3^

From a clinical and public health perspective, the Brazilian experience with PCV10 shows that vaccines are not static in their effects. The initial impact was significant: a one-third reduction in hospitalizations for childhood pneumonia. However, this protection did not remain uniform over time, as the phenomenon of serotype replacement brought new challenges. The most striking example is serotype 19A, which is not included in PCV10 and has become a common cause of pneumonia, often associated with severe cases and antimicrobial resistance. This means that, in clinical practice, pediatricians and primary care physicians should remain aware that children vaccinated with PCV10 are still at risk for pneumonia caused by nonvaccine serotypes. In severe cases, especially those of hospitalization, it is necessary to consider the possibility of infection by serotype 19A and adjust therapeutic management to local patterns of resistance. This reinforces the importance of integrating clinical reasoning into epidemiological surveillance, given that the prevalence of serotypes may vary across regions and change rapidly.^6^

For policymakers and health authorities, the lesson is equally clear: the success of a vaccine depends on the ability to monitor the dynamics of disease. Transitioning to higher-valency vaccines, such as PCV13, the 15-valent PCV, and the 20-valent PCV, offers broader protection and represents an opportunity to resume the reductions observed in the first decade after PCV10 was introduced. International experience confirms this path. Countries that adopted PCV13 saw marked declines in the prevalence of serotype 19A, including cases of invasive disease. This effect is not limited to reducing hospitalizations but also impacts antimicrobial resistance, given that 19A is known for presenting higher resistance profiles. 

Another important aspect for practice is understanding that continuous surveillance should not be seen as the sole responsibility of central public health authorities. Physicians, hospitals, and laboratories play an active role in feeding information systems and identifying changes in the clinical profile of diseases. Accurate reporting and collection of microbiological data are essential components to guide effective vaccination policies. 

Finally, the discussion on pneumococcal serotypes illustrates a broader point: immunization strategies must be constantly updated to respond to the dynamic behavior of microorganisms. Scientific advances have allowed the development of vaccines covering 15 to 20 serotypes, with the potential to include not only 19A but also emerging serotypes such as 22F and 33F. Incorporating these options is not just a technical decision but also a strategic one, capable of preventing setbacks and ensuring long-term protection for children. 

In summary, the lesson is that pneumococcal vaccination should be regarded as a constantly evolving process. Although PCV10 has brought concrete benefits, the current epidemiological reality demands expansion of the vaccine arsenal. Updating immunization strategies, adopting higher-valency vaccines, and maintaining active surveillance are fundamental steps to consolidate protection against severe pneumonia and ensure that the progress achieved in the past decade is not compromised by emerging serotypes. 
